# A neural network for prediction of risk of nosocomial infection at intensive care units: a didactic preliminary model

**DOI:** 10.31744/einstein_journal/2020AO5480

**Published:** 2020-11-12

**Authors:** Beatriz Nistal-Nuño

**Affiliations:** 1 Complejo Hospitalario Universitario de Santiago de Compostela Santiago de Compostela Spain Complejo Hospitalario Universitario de Santiago de Compostela, Santiago de Compostela, Spain.

**Keywords:** Artificial neural network, Nosocomial infection, Intensive care units, APACHE, Artificial intelligence

## Abstract

**Objective:**

To propose a preliminary artificial intelligence model, based on artificial neural networks, for predicting the risk of nosocomial infection at intensive care units.

**Methods:**

An artificial neural network is designed that employs supervised learning. The generation of the datasets was based on data derived from the Japanese Nosocomial Infection Surveillance system. It is studied how the Java Neural Network Simulator learns to categorize these patients to predict their risk of nosocomial infection. The simulations are performed with several backpropagation learning algorithms and with several groups of parameters, comparing their results through the sum of the squared errors and mean errors per pattern.

**Results:**

The backpropagation with momentum algorithm showed better performance than the backpropagation algorithm. The performance improved with the xor. README file parameter values compared to the default parameters. There were no failures in the categorization of the patients into their risk of nosocomial infection.

**Conclusion:**

While this model is still based on a synthetic dataset, the excellent performance observed with a small number of patterns suggests that using higher numbers of variables and network layers to analyze larger volumes of data can create powerful artificial neural networks, potentially capable of precisely anticipating nosocomial infection at intensive care units. Using a real database during the simulations has the potential to realize the predictive ability of this model.

## INTRODUCTION

An important cause of morbidity and mortality are nosocomial infections, implying a significant burden on patients and hospitals.^(^^1^^)^ These complications are highly associated with stay at intensive care units (ICU).^(^^2^^–^^4^^)^ Studies have shown that ICU-acquired nosocomial infections have an important effect on mortality.^(^^5^^–^^7^^)^

Identification of many nosocomial infections is currently conducted via specimen culture, which can take a few days. Consequently, an overuse of broad-spectrum antibiotics is the current treatment, thus treating potential cases who may not suffer from these complications and resulting in the potential risk of evolving antibiotic-resistant strains.^(^^7^^)^

Surveillance of nosocomial infections, together with appropriate prevention measures, can decrease infection rates and improve patient safety. It is crucial for infection control professionals to predict and measure the risk of infection at their hospitals, contributing to planning and assessment of infection control programs.^(^^1^^)^ There are national or regional programs to support hospitals in reducing the risk of nosocomial infections, *e.g.* , the Japanese Nosocomial Infection Surveillance (JANIS) system in Japan, and the Healthcare-Associated Infections Surveillance Network in Europe (HAI-Net). The World Health Organization (WHO) recommends that risk prevention of nosocomial infections must also be supported by hospital programs.

If predictive tools could be evolved to reveal those most likely to contract these nosocomial infections, then a reduced risk of resistant strains would ensue, leading to significant savings regarding mortality and treatment expenses.

Prediction of nosocomial infections is generally subjective, dependent on interpretation, in which clinicians with widely varying experiences assess patient conditions. This dependency restricts the ability to obtain accurate correlations between risk factors and outcome predictions. Thus, it is a matter of experience the value applied to any given situation.^(^^8^^)^ However, protocols derived from the national or regional programs help ensuring standardization of definitions orienting diagnosis and treatment in ICU, in addition to institutional protocol guidance.

The application of artificial neural networks (ANN) in outcome prediction has become increasingly prevalent in physiological modeling^(^^8^^,^^9^^)^ and several medical fields,^(^^7^^,^^10^^–^^17^^)^ due to the ability of the ANN to learn and improve.

Artificial neural networks are artificial intelligence models built based on biological neural systems. These ANN are adaptive systems which are increasingly used for prediction and are especially adequate for predictions of classification events.

### Literature comparing different alternatives

The study by Burke et al.,^(^^18^^)^ compared ANN to other statistical methods for medical outcome prediction, particularly survival prediction. They found that using just the tumor-node-metastasis (TNM) variables, both the backpropagation neural network and the probabilistic neural network were significantly more accurate than the pathological TNM stage system.^(^^18^^)^

Eftekhar et al.,^(^^19^^)^ contrasted the performance of ANN and multivariable logistic regression (LR) models in prediction of outcomes in head trauma. They found that ANN significantly outperformed LR in the fields of discrimination and calibration.^(^^19^^)^ DiRusso et al.,^(^^20^^)^ developed an ANN to predict pediatric trauma death and compared it with LR. The ANN model yielded excellent discrimination and calibration exceeding that of LR.^(^^20^^)^ Erguzel et al.,^(^^21^^)^ used the two mentioned methods to create a model dichotomizing opioid-dependent patients and control subjects. The ANN classifier outperformed the LR.^(^^21^^)^ Liew et al.,^(^^22^^)^ retrospectively analyzed the prevalence and risk factors of gallbladder disease using LR and ANN among obese patients. Artificial neural networks, constructed with backpropagation algorithm, were trained to predict the risk of gallbladder disease. Artificial neural networks demonstrated better average classification rate, and lower type II errors than LR.^(^^22^^)^

The use of ANN in this particular setting has not been widely studied. A few related studies have been carried out successfully on trauma patients,^(^^7^^,^^10^^)^ whereas other recent studies have concentrated on mortality prediction.^(^^7^^–^^9^^,^^23^^)^

### Supervised learning in artificial neural networks

The formal model underlying neural computation in ANN is a parallel and directed graph in which the nodes are associated to models of local calculi, and the links represent the interconnections between these local calculi.^(^^24^^)^

The output of each node can generally be expressed as the weighted sum of the coordinates of the input array (x) multiplied by the coordinates corresponding to the array of weights (w), as Equation 1 .

(Equation 1) yj*(t)=Σi=1 to M[wji(t) *xi(t)] for j=1,2, …N0

The result of this sum afterwards passes through a decision function, that produces the final outputs *yj(t)* ( Equation 2 ).

(Equation 2) yj(t)=u[yj*(t)]

By its derivable character (to calculate the new weights), the sigmoid function is usually used as the decision function, which provides an activation value in the range [0.1]. The local function is adjusted through learning processes. By using an algorithm methodology known as supervised learning, the algorithm varies the value of the weights connecting neuronal units according to deviations from a training set of data, which specifies the activation values of the output units corresponding to a set of input observations (labeled data).^(^^24^^)^ The problems solved with supervised learning are based on adaptive numerical classifiers.^(^^24^^)^

### Backpropagation in multilayer networks

This function is especially adequate for propagating the observed error to hidden layers, which have not available the desired outputs for those hidden neurons. This ANN is trained with supervised learning type of backpropagation.^(^^24^^)^ In this way, the errors are propagated backward.^(^^24^^)^

## OBJECTIVE

The purpose of this artificial neural network is to calculate the risk of nosocomial infection for the intensive care unit patients evaluated. This is from the perspective of a simulated model, based on synthetic datasets derived from an actual database for the purpose of demonstration. It is intended to present a preliminary artificial neural network in this medical context, and show its construction and step-by-step analyses, so that adjustments and improvements can be easily incorporated to meet the needs of specific users. It is explained how to propose and solve this model through an artificial neural network utilizing supervised learning algorithms. The differences in behavior of the algorithms are analyzed in terms of the parameter variations, trying to find an optimal design.

## METHODS

### Selection of factors associated with the development of nosocomial infection

The selection of the risk factors for the elaboration of the synthetic datasets was based on the factors revealed by Suka et al.^(^^1^^)^ This study was grounded on data acquired from the ICU component of the JANIS system to elucidate factors associated with the development of nosocomial infections and to determine infection rates for benchmarking. These factors were selected based on a multivariate analysis. They determined hazard ratios (HR) with 95% confidence intervals for the various factors selected.

The generation of the synthetic datasets was based on the revealed HR of the factors associated to the development of nosocomial infections.^(^^1^^)^ Data were constructed derived from the information given by Barraclough et al.,^(^^25^^)^ with each binary value of each variable obtained from a probability distribution according to its HR, depending on the classification of that patient. The training and validation datasets follow a realistic distribution of the values involved.

### Spreadsheet composition

All patients share several attributes that can be quantified. The columns in the spreadsheet represent these attributes, which distinguish the patients. Binary variables are used to represent if each patient possesses each attribute. These binary variables are used to describe the activity of the input and output neuronal units.

In the spreadsheets, each row, or pattern, stands for a different ICU patient. These data are imported into the Java Neural Network Simulator (JavaNNS)^(^^26^^)^ to execute the simulations. This work analyzes how the JavaNNS^(^^26^^)^ learns to represent and categorize these patients, based on their selected attributes, into the categories of present and absent risk of nosocomial infections. That is, a patient who is at risk of suffering or not from nosocomial infections.

Several major patient-level risk factors associated with the development of nosocomial infections were selected, as described above, for characterization of the patients, according to table 1 .

**Table 1:** Major patient-level risk factors selected associated with the development of nosocomial infections

Factor		
Sex		Male=1; female=0
Age		
	Age 16-44	Yes=1; no=0
	Age 45-64	Yes=1; no=0
	Age 65-74	Yes=1; no=0
	Age ≥75	Yes=1; no=0
Severity of illness, APACHE II score		
	APACHE II score 0-10	Yes=1; no=0
	APACHE II score 11-20	Yes=1; no=0
	APACHE II score ≥21	Yes=1; no=0
Operation (both elective and urgent)		Yes=1; no=0
Device use		
	Ventilator use	Yes=1; no=0
	Urinary catheter	Yes=1; no=0
	Central venous catheter	Yes=1; no=0

APACHE II: Acute Physiology and Chronic Health disease Classification System II.

Since these factors were selected based on the study of Suka et al.,^(^^1^^)^ who utilized the JANIS database, this fictitious ICU would have the same characteristics: patients aged 16 years or more, who had remained at the ICU for 48 to 1,000 hours, who had not been moved to another ICU, and had not been infected within 2 days after ICU admission.^(^^1^^)^ The categorization provided by the ANN is into the following output unit: risk of nosocomial infections – yes=1; no=0.

Each row represents a set of input and output units. Two different spreadsheets were created with these characteristics. One has 15 patients or patterns that was used for training the network.^(^^27^^)^ This training set was chosen to be of a size of 15 times the number of classes to obtain.^(^^28^^)^ The other spreadsheet has six patients or patterns that was used for validation of the network.^(^^27^^)^ This validation set was chosen to be of a size of six times the number of classes to obtain.^(^^28^^)^

### Elaboration of the pattern files of Java Neural Network Simulator

To create the JavaNNS pattern files, the MATLAB® program was used, version 7.5.0.342 (R2007b). MATLAB® has this functionality, which is used by typing the command “xls2nns([])”. A new file is then created with the same name as the spreadsheet but with a .pat extension. The two pattern files created, ICU trainingdata_NI.pat^(^^27^^)^ and ICU validationdata_NI.pat,^(^^27^^)^ are used for training and validation of the network respectively. Validation is used to determine the performance of the ANN on patterns that are not trained during learning.^(^^24^^)^

### Neural network simulations

**Figure 1:**
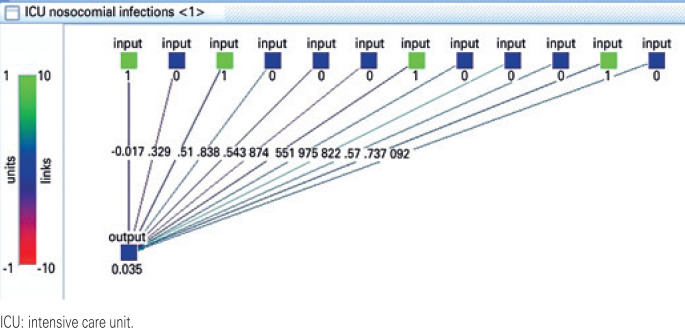
The utilized neuronal network architecture designed specifically for this problem in Java Neural Network Simulator. The state of the network here shows a trained artificial neural network with the output prediction of the network for one of the validation patterns

A multilayer perceptron ANN, a feed-forward network without shortcut connections is used. In JavaNNS, going to Tools/Create/Layers, the width is changed to be 12, the number of neuronal input units. Again, in Tools/Create/Layers, the width is changed to be 1, the number of output units. Afterwards, the network is connected feed forward.^(^^26^^)^ The resulting network is shown in figure 1 .

The initializing function is set to random weights and the *Init* button is pressed, setting the weights to random values.^(^^26^^)^

### The utilized learning functions and learning-parameters

Among the supervised learning algorithms, the backpropagation type was used. There are several backpropagation algorithms provided with JavaNNS. The backpropagation and backpropagation with *momentum* algorithms^(^^24^^)^ are employed to train this network.

The values of the parameters used in the simulations are shown in table 2 . These are obtained from the ones given in the xor. README file of the JavaNNS examples, and the ones given by default in JavaNNS.^(^^24^^,^^26^^)^ The parameters in the xor. README file are some parameters that Fischer et al.,^(^^26^^)^ used to train successfully the XOR-problem on a neural network. This XOR-problem ANN has some similarities to the one constructed in this research. Fischer et al.,^(^^26^^)^ suggested the number of cycles indicated in table 2 as the ones they needed to train the XOR-problem network successfully. These parameters were not obtained with extensive studies of statistical significance, they are given in JavaNNS as hints to start training sessions.^(^^26^^)^

**Table 2:** Parameters values used for all models of combinations of learning functions and parameter sets

Learning function	η	*d*_max_	μ	*c*	Cycles
Backpropagation with the xor. README file values	2.0	0.1			2,000
Backpropagation with the default values	0.2	0.1			2,000
Backpropagation with momentum with the xor. README file values	3.0	0.1	0.8	0.1	100
Backpropagation with momentum with the default values	0.2	0.1	0.5	0.1	100

### Simulations

Since there is a random component in the experiment, ten simulations are performed for each of the learning algorithms and set of parameters, comparing their results through the sum of the squared errors (SSE) of the Log and Error graph windows.^(^^26^^)^ The results are compared with previous parameters and between the different trials.

Using the validation set, in the Error graph window appears simultaneously an additional line of pink color that corresponds to the error of the validation set. It is utilized the SSE, the sum of the quadratic differences between the target output and the real output for all output units in all training or validation patterns. The Log window shows during training the SSE values in the validation and training sets.^(^^24^^)^ The SSE from the Log window are afterwards divided manually by the number of patterns in each set ( Table 3 ).

**Table 3:** Mean squared error per pattern for each one of the learning functions and sets of parameters, shown for each one of the sets of patterns. These are obtained from the sum of the squared errors shown in the Log window corresponding to the last trial and dividing by the number of patterns in each set

Learning function	MSE	
	Training set	Validation set
Backpropagation with the xor. README file values	0.0	0.02760293
Backpropagation with the default values	0.0	0.01951062181
Backpropagation with momentum with the xor. README file values	0.0	0.0
Backpropagation with momentum with the default values	0.0	0.01153508821

MSE: mean squared error.

Once finished the training, in the Updating window, the training and validation patterns are passed one by one. Each pattern is visualized by colors and numerically, in all neurons of the network, to observe if the network succeeded or failed with each input pattern. The trained ANN is evaluated also by a confusion matrix.

**Figure 2:**
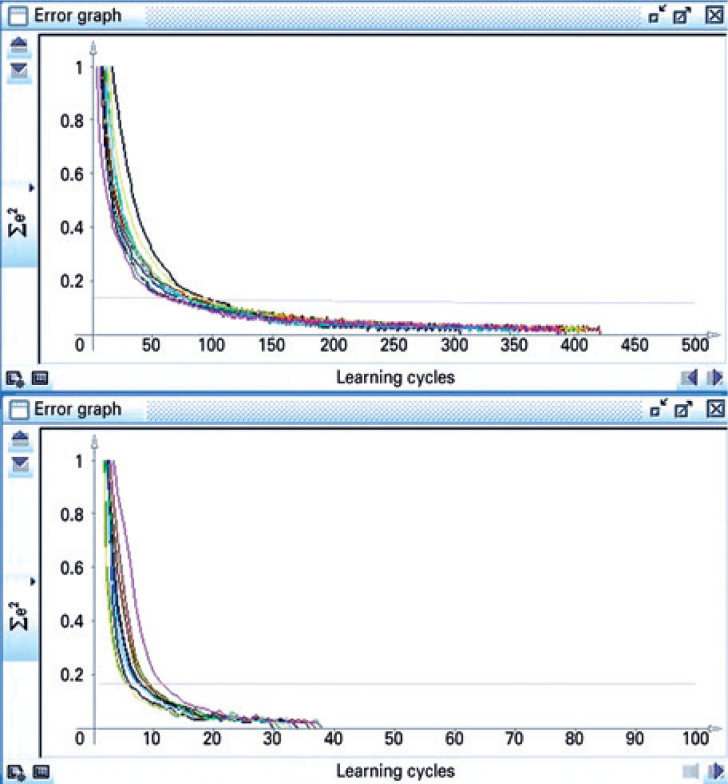
Ten simulations performed in Java Neural Network Simulator with the backpropagation learning function, comparing their results through the sum of the squared errors in the Error graph window. The pink line corresponds to the sum of the squared errors of the validation set during the last trial. Bottom: with the xor. README file parameter values. Top: with the default parameter values

**Figure 3:**
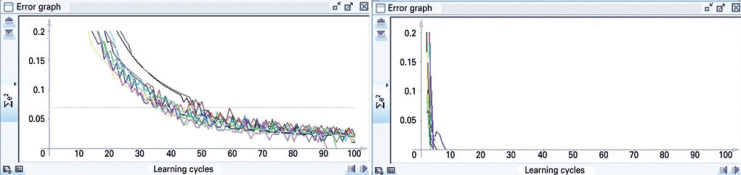
Ten simulations performed in Java Neural Network Simulator with the backpropagation with momentum learning function, comparing their results through the sum of the squared errors in the Error graph window. The pink line corresponds to the sum of the squared errors of the validation set during the last trial. On the right: with the xor. README file parameter values. On the left: with the default parameter values

**Figure 4:**
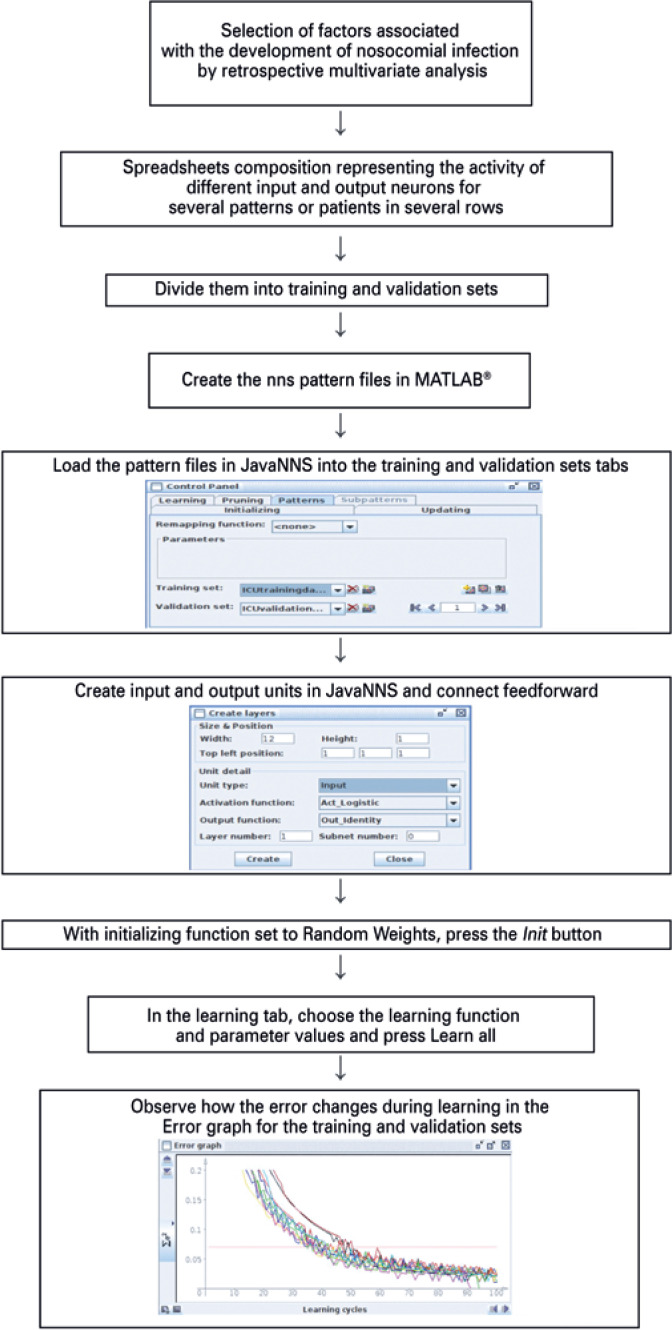
Flowchart for supervised learning in Java Neural Network Simulator

The update mode topological order is used.^(^^24^^)^ Pressing Learn all in the learning tab cycles through all PATTERNS, for the number of times (cycles) specified. For the ten trials, the buttons *Init* and Learn all are pressed once for each trial. The graphs accumulate in the Error graph window in different colors ( Figures 2 and 3 ). A flowchart summarizing the methods is displayed in figure 4 .

## RESULTS

### Backpropagation learning function

The backpropagation algorithm had the following parameters: learning parameter η, indicating the step width of the gradient descent, and *dmax* , corresponding to the maximum difference between a target output and an actual output which is tolerated.^(^^24^^)^

### Backpropagation with momentum learning function

The backpropagation with momentum algorithm had the following parameters:^(^^24^^)^ learning parameter η, as mentioned; *μ,* momentum term, indicating the amount of the previous weight adjustment (relative to 1) which was added to the current change;^(^^24^^)^ *c,* flat spot elimination value, a constant added to the derivative of the activation function so the network could pass flat spots of the error surface,^(^^24^^)^ and *dmax,* as mentioned.

### Errors table and comments of the results

In figure 2 , bottom, the SSE from the training patterns disappeared in all trials before 40 cycles, well before the maximum of 2,000 cycles. The MSE of the validation set was 0.02760293, which was a very small error. At 2,000 cycles this value stayed the same, at this MSE the network performed best and was when the training should be stopped.

In figure 2 , top, the SSE decreased progressively until reaching 0.0 in all trials before 450 cycles. The pink line reached its minimum MSE, also a very small error. In comparison to figure 2 , bottom, the default values showed here a worse performance.

In figure 3 , right, the SSE was 0.0 at before ten cycles for all trials. The pink line MSE was 0.0. This backpropagation with momentum algorithm showed improvement in comparison to the backpropagation algorithm.

In figure 3 , left, the SSE decreased progressively until becoming 0.0 at 100 cycles. The pink line MSE was 0.011535088, a very small value. The performance with the default values was worse than with the xor. README file values for this backpropagation with momentum algorithm. For the default parameter values, this backpropagation with momentum algorithm showed better performance than the backpropagation algorithm.

In table 4 , it is presented the classification obtained after training for the 15 training and six validation patterns showing the successes and failures. A pattern was classified correctly into present risk of nosocomial infections if the output unit activation was ≥0.6. A pattern was classified correctly into absent risk of nosocomial infections if the output unit activation was ≤0.4.

**Table 4:** Classification for the 15 training and six validation patterns, obtained in the Updating tab after training with the training patterns

Learning function	Training patterns		Validation patterns	
	Successes	Failures	Successes	Failures
Backpropagation with the xor. README file values	15	0	6	0
Backpropagation with the default values	15	0	6	0
Backpropagation with momentum with the xor. README file values *	15	0	6	0
Backpropagation with momentum with the default values	15	0	6	0

* With these function and parameters, the error of the units was particularly small, almost nil.

There were no failures in the categorization of the patients into their corresponding risk of nosocomial infections. The backpropagation with momentum with the xor. README file values showed an almost nil error. The network performed best with the backpropagation with momentum algorithm with the xor. README file values.

### Evaluation by a confusion matrix

The most likely prediction state for each pattern was then compared with the true value of the output unit activation for the particular case. The confusion matrix supplies the total number of cases in 21 situations. If the network was performing well, then the entries along the main diagonal would be large, compared to those off of it. This is observed in table 5 .

**Table 5:** Confusion matrix of predicted and actually occurred outcome of states for risk of nosocomial infection in 21 cases of the training and validation datasets

Risk of nosocomial infections		Predicted	
		Present risk of nosocomial infections	Absent risk of nosocomial infections
**Actual**	Present risk of nosocomial infections	12	0
	Absent risk of nosocomial infections	0	9

## DISCUSSION

The results revealed relative variations in performance for the different combinations of learning algorithms and parameter sets. The function of backpropagation with momentum showed the best results. The results were better with the xor. README file parameter values. However, as stated by Fischer et al *.,* ^(^^26^^)^ these parameters should not be cited as optimal or used in comparisons of network simulators.

The ability of this ANN to predict those likely to contract nosocomial infections is 100% accurate on the basic features given. If several attributes are included for characterization as input variables and/or numerous classes are included as output variables, it would be necessary to include a big amount of patterns. If after testing the network for its successes and failures with each of the training and validation patterns, a high percentage of successes is not obtained, then it could be necessary to add one or several layers of hidden neurons. Additionally, other kind of backpropagation learning functions could be used in JavaNNS, such as the Quickprop or the Resilient propagation algorithms.

The distribution of risk factors for nosocomial infections differs broadly according to hospital and time. Because of these variations, data from different regions may be of a diverse nature with differences in the incidence of nosocomial infections. Failure to adjust adequately for the particular region and time could lead to false conclusions. It is recommended to use multivariate analyses retrospectively to reveal the risk factors associated with the outcome under study. Float variables can be used also with JavaNNS. In fact, using continuous input data would reduce the number of input neurons and would simplify network architecture. It is recommended to use as few variables as possible.^(^^8^^)^

In this study, nosocomial infections were not classified by infection site. This research could inform researchers to be able to expand this project, and the spreadsheets could be prepared by infection site. Once the model is set within a context of real data, it should be evaluated with, at least, a validation set of patterns of real cases. This will need to be validated and tested with a sufficient number of actual patient patterns, relying on large volumes of data. In this medical context, the datasets should have hundreds or thousands of pattern samples. In the case of a very reduced dataset, a cross-validation scheme should be used for network validation. Moreover, other types of predictive methods could be tested to compare the results to the ANN. The only study found in the literature applied to a similar clinical setting, addressing some of these limitations, is the study by Chang et al.^(^^29^^)^

## CONCLUSION

This article proposes a preliminary method for predicting risk of nosocomial infection at intensive care units, adopting artificial intelligence as artificial neural networks to achieve improvements. This model is used to show its design and step-by-step analyses, using a synthetic dataset derived from the Japanese Nosocomial Infection Surveillance system. While this model is still based on a synthetic dataset, the excellent performance observed with a small number of patterns suggests using higher numbers of variables and network layers, to analyze larger volumes of data, can create powerful artificial neural networks, potentially capable of precisely anticipating nosocomial infection at intensive care units.
