# Supplementary material for: A neural network for prediction of risk of nosocomial infection at intensive care units: a didactic preliminary model
Source: Einstein (Sao Paulo). 2020 Nov 12;18:eAO5480. doi: 10.31744/einstein_journal/2020AO5480 (PMC7664827; doi:10.31744/einstein_journal/2020AO5480)
Supplement: Translation

# Translation: Uma rede neural de previsão de riscos de infecção nosocomial em unidades de cuidado intensivo: um modelo preliminar didático

**Authors:** Beatriz Nistal-Nuño

**Affiliations:** 1 Santiago de Compostela Spain Complejo Hospitalario Universitario de Santiago de Compostela, Santiago de Compostela, Spain.

**Keywords:** Rede neural artificial, Infecção hospitalar, Unidades de terapia intensiva, APACHE, Inteligência artificial

## Abstract

**Objetivo:**

Propor um modelo preliminar de inteligência artificial, baseado em redes neurais artificiais, para previsão do risco de infecção hospitalar em unidades de cuidado intensivo.

**Métodos:**

Foi usada uma rede neural artificial, que utiliza aprendizagem supervisionada. A geração dos conjuntos de dados baseia-se em dados derivados do sistema *Japanese Nosocomial Infection Surveillance* . Estudamos como o *Java Neural Network Simulator* aprende a categorizar esses pacientes para prever o respectivo risco de infecção hospitalar. As simulações são realizadas com diferentes algoritmos de aprendizagem por retropropagação e diversos grupos de parâmetros, comparando-se os resultados com base na soma dos erros quadráticos e erros médios por padrão.

**Resultados:**

O algoritmo de retropropagação com *momentum* mostrou desempenho superior ao do algoritmo de retropropagação. O desempenho foi melhor com os valores de parâmetros do arquivo xor. README em comparação aos parâmetros *default* . Não houve falhas na categorização de pacientes quanto ao respectivo risco de infecção hospitalar.

**Conclusão:**

Embora esse modelo se baseie em um conjunto de dados sintéticos, o excelente desempenho observado com um pequeno número de padrões sugere que o uso de números maiores de variáveis e camadas de rede para analisar volumes maiores de dados pode criar redes neurais artificiais poderosas, possivelmente capazes de prever com precisão o risco de infecção hospitalar em unidades de cuidado intensivo. O uso de um banco de dados real durante as simulações torna possível a realização da capacidade preditiva desse modelo.

## INTRODUÇÃO

As infecções hospitalares são importante causa de morbidade e mortalidade, levando a um ônus significativo para pacientes e hospitais.^(^^1^^)^Essas complicações são associadas à internação em unidades de terapia intensiva (UTI).^(^^2^^–^^4^^)^Estudos mostram que as infecções hospitalares contraídas em UTI têm efeito considerável na mortalidade.^(^^5^^–^^7^^)^

Muitas infecções hospitalares são atualmente identificadas por meio da cultura de amostras, e isso pode levar alguns dias. Por isso, o tratamento atual é o uso excessivo de antibióticos de amplo espectro, já que nem todos os casos tratados chegam a apresentar efetivamente essas complicações, o que leva a um possível risco de desenvolvimento de cepas resistentes a antibióticos.^(^^7^^)^

A vigilância de infecções hospitalares, combinada a medidas preventivas apropriadas, pode reduzir as taxas de infecção e melhorar a segurança do paciente. É fundamental que os profissionais de controle de infecções possam prever e mensurar o risco de infecção nos hospitais e contribuir para o planejamento e a avaliação de programas de controle.^(^^1^^)^Existem programas nacionais ou regionais para auxiliar os hospitais na redução do risco de infecções hospitalares, como *Japanese Nosocomial Infection Surveillance* (JANIS), no Japão, e a *Healthcare-Associated Infections Surveillance Network* (HAI-Net), na Europa. A Organização Mundial da Saúde (OMS) recomenda que a previsão do risco de infecções hospitalares também seja promovida no âmbito dos programas hospitalares.

Se for possível criar ferramentas que revelem quem são as pessoas com maior risco de contrair infecções hospitalares, poderemos reduzir o risco de desenvolvimento de cepas resistentes, levando a uma diminuição considerável da mortalidade e das despesas com tratamento.

A previsão de infecções hospitalares é normalmente subjetiva e depende de interpretação, com grande variação na experiência dos clínicos que avaliam as condições dos pacientes. Essa dependência limita a capacidade de se obterem correlações precisas entre fatores de risco e a previsão de desfechos. Porém, a definição do valor aplicado a uma determinada situação é uma questão de experiência.^(^^8^^)^No entanto, protocolos derivados dos programas nacionais ou regionais ajudam a assegurar a padronização de definições para guiar o diagnóstico e o tratamento em UTI, complementando a orientação dos protocolos institucionais.

A aplicação de redes neurais artificiais (RNAs) na previsão de desfechos já é cada vez mais prevalente na modelagem fisiológica^(^^8^^,^^9^^)^e em diferentes áreas médicas,^(^^7^^,^^10^^–^^17^^)^devido à capacidade das RNAs de aprender a se aprimorar.

As RNAs são modelos de inteligência artificial construídos com base em sistemas neurais biológicos. Essas redes constituem sistemas adaptativos, usados cada vez mais para fins de previsão e especialmente adequados para previsões de eventos de classificação.

### Comparação de diferentes alternativas na literatura

O estudo de Burke et al.,^(^^18^^)^comparou as RNAs a outros métodos estatísticos para previsão de desfechos médicos e, especificamente, de sobrevida. Os autores observaram que, usando apenas as variáveis tumor-linfonodo-metástase (TNM), tanto a rede neural de retropropagação quanto a rede neural probabilística foram consideravelmente mais precisas que o sistema TNM de estadiamento patológico.^(^^18^^)^

Eftekhar et al.,^(^^19^^)^contrastaram o desempenho da RNA e modelos de regressão logística (RL) multivariada na previsão de desfechos em traumatismo craniano. Encontraram um desempenho significativamente melhor da RNA em comparação à RL nas áreas de discriminação e calibração.^(^^19^^)^DiRusso et al.,^(^^20^^)^desenvolveram uma RNA para prever a mortalidade por trauma pediátrico e a compararam com a RL. O modelo da RNA demonstrou discriminação e calibração excelentes, superiores às da RL.^(^^20^^)^Erguzel et al.,^(^^21^^)^usaram os dois métodos para criar um modelo de dicotomização de pacientes opioide-dependentes e controles. O classificador baseado em RNA teve desempenho superior à RL.^(^^21^^)^Liew et al.,^(^^22^^)^analisaram retrospectivamente a prevalência e os fatores de risco de doença da vesícula biliar usando RL e RNA em pacientes obesos. As RNAs, construídas com algoritmo de retropropagação, foram treinadas para prever o risco de doença da vesícula biliar. Elas demonstraram uma taxa de classificação média superior e menos erros tipo II quando comparadas à RL.^(^^22^^)^

O uso de RNAs nesse cenário em particular ainda não foi amplamente estudado. Alguns poucos estudos relacionados foram conduzidos com êxito em pacientes com traumatismo,^(^^7^^,^^10^^)^enquanto outros estudos recentes concentraram-se na previsão de mortalidade.^(^^7^^–^^9^^,^^23^^)^

### Aprendizagem supervisionada em redes neurais artificiais

O modelo formal por trás da computação neural nas RNAs é um gráfico paralelo e dirigido, no qual os nós são associados a modelos de cálculos locais, e os *links* representam as interconexões entres esses cálculos locais.^(^^24^^)^

A saída ( *output* ) de cada nó é normalmente expressa como a soma ponderada das coordenadas do *input array* (x), multiplicada pelas coordenadas correspondentes ao *array* de pesos (w), conforme Equação 1 .

(Equação 1) yj*(t)=Σi=1 a M[wji(t)*xi(t)] para j=1,2, …N0

O resultado dessa soma, depois, passa por uma função de decisão que produz as saídas finais *yj(t)* ( Equação 2 ).

Equação 2 yj(t)=u[yj*(t)]

Com base no caractere derivável (para calcular os novos pesos), a função sigmoide é normalmente usada como de decisão e fornece um valor de ativação dentro do intervalo [0,1]. A função local é ajustada por processos de aprendizagem. Usando uma metodologia de algoritmos conhecida como aprendizagem supervisionada, o algoritmo varia o valor dos pesos que conectam as unidades neuronais de acordo com os desvios de um conjunto de dados de treinamento, que especificam os valores de ativação das unidades de saída correspondentes a uma série de observações de entrada (dados rotulados).^(^^24^^)^Os problemas solucionados com aprendizagem supervisionada baseiam-se em classificadores numéricos adaptativos.^(^^24^^)^

### Retropropagação em redes multicamadas

Essa função é especialmente adequada para a propagação do erro observado para as camadas ocultas, já que não temos disponíveis as saídas desejadas para esses neurônios ocultos. Essa RNA é treinada pela aprendizagem supervisionada do tipo retropropagação.^(^^24^^)^Dessa forma, os erros são propagados de trás para a frente.^(^^24^^)^

## OBJETIVO

O objetivo dessa rede neural artificial é calcular o risco de infecção hospitalar dos pacientes da unidade de terapia intensiva avaliada. Isso é feito a partir de um modelo simulado, baseado em conjuntos de dados sintéticos derivados de um banco de dados real, para fins de demonstração. A intenção é apresentar uma rede neural artificial preliminar nesse contexto médico e demonstrar a construção e as análises passo a passo, facilitando a incorporação de quaisquer ajustes e melhorias que se façam necessários para atender às necessidades de usuários específicos. Além disso, mostramos como é possível propor e solucionar esse modelo por meio de uma rede neural artificial baseada em algoritmos de aprendizagem supervisionada. Analisamos as diferenças no comportamento dos algoritmos com a variação dos parâmetros, tentando chegar ao desenho ideal.

## MÉTODOS

### Seleção de fatores associados ao desenvolvimento de infecção hospitalar

A seleção dos fatores de risco para elaboração dos conjuntos de dados sintéticos baseou-se nos fatores revelados por Suka et al.,^(^^1^^)^que usaram dados adquiridos do componente de UTI do sistema JANIS. Os autores analisaram tais dados para elucidar fatores associados ao desenvolvimento de infecções hospitalares e determinar taxas de infecção para *benchmarking* . Esses fatores foram selecionados com base em uma análise multivariada. Foram determinadas as *hazard ratios* (HR) com intervalos de confiança de 95% para os diversos fatores selecionados.

A geração dos conjuntos de dados sintéticos baseou-se nos HR revelados dos fatores associados ao desenvolvimento de infecções hospitalares.^(^^1^^)^Os dados foram construídos com base nas informações fornecidas por Barraclough et al.,^(^^25^^)^com o valor binário de cada variável obtido de uma distribuição de probabilidades, de acordo com o respectivo HR, dependendo da classificação do paciente em questão. Os conjuntos de dados de treinamento e validação seguem uma distribuição realista dos valores envolvidos.

### Composição das planilhas

Todos os pacientes compartilham diversos atributos que podem ser quantificados. As colunas na planilha representam esses atributos, que diferenciam os pacientes. As variáveis binárias são usadas para representar se cada paciente apresenta cada um desses atributos. Essas variáveis binárias são usadas para descrever a atividade das unidades neuronais de entrada e saída.

Nas planilhas, cada linha, ou padrão *(pattern)* , representa um paciente de UTI diferente. Esses dados são importados no Java Neural Network Simulator (JavaNNS)^(^^26^^)^para que sejam executadas as simulações. Esse trabalho analisa como o JavaNNS^(^^26^^)^aprende a representar e a categorizar esses pacientes com base em atributos selecionados, colocando-os nas categorias de risco de infecções hospitalares presente e ausente, ou seja, se um paciente tem risco ou não de contrair infecções hospitalares.

Diversos fatores de risco importantes relativos ao paciente e associados ao desenvolvimento de infecções hospitalares foram selecionados, para caracterização dos pacientes, conforme tabela 1 .

**Tabela 1:** Fatores de risco importantes relativos ao paciente e associados ao desenvolvimento de infecções hospitalares selecionados

Fator		
Sexo		Masculino=1; feminino=0
Idade		
	Idade 16-44	Sim=1; não=0
	Idade 45-64	Sim=1; não=0
	Idade 65-74	Sim=1; não=0
	Idade ≥75	Sim=1; não=0
Gravidade da doença, escore APACHE II		
	Escore APACHE II 0-10	Sim=1; não=0
	Escore APACHE II 11-20	Sim=1; não=0
	Escore APACHE II ≥21	Sim=1; não=0
Cirurgia (tanto eletiva quanto urgente)		Sim=1; não=0
Uso de dispositivos		
	Uso de ventilador	Sim=1; não=0
	Sonda urinária	Sim=1; não=0
	Acesso venoso central	Sim=1; não=0

APACHE II: *Acute Physiology and Chronic Health disease Classification System II* .

Como esses fatores foram selecionados com base no estudo de Suka et al.,^(^^1^^)^que utilizaram o banco de dados do JANIS, essa UTI fictícia teria as mesmas características: pacientes de 16 anos ou mais, com tempo de internação na unidade de 48 a 1.000 horas, que não tivessem sido transferidos para outra UTI, e não tivessem sido infectados nos primeiros 2 dias após a internação na unidade.^(^^1^^)^A categorização fornecida pela RNA é na seguinte unidade de saída: risco de infecções hospitalares – sim=1; não=0.

Cada linha representa um conjunto de unidades de entrada e saída. Duas planilhas diferentes foram criadas com essas características. Uma tem 15 pacientes ou padrões e foi usada para treinamento da rede.^(^^27^^)^Esse conjunto de treinamento foi escolhido de modo que tivesse um tamanho 15 vezes maior que o número de classes a serem obtidas.^(^^28^^)^A outra planilha tem seis pacientes ou padrões e foi usada para validação da rede.^(^^27^^)^Esse conjunto de validação foi escolhido de modo que tivesse um tamanho seis vezes maior que o número de classes a serem obtidas.^(^^28^^)^

### Elaboração dos arquivos padrão do *Java Neural Network Simulator*

Para criar os arquivos padrão do JavaNNS, usou-se o programa MATLAB®, versão 7.5.0.342 (R2007b). O MATLAB® tem essa funcionalidade, que pode ser usada digitando-se o comando “xls2nns([])”. Cria-se, então, um novo arquivo, com o mesmo nome da planilha, mas com a extensão .pat. Os dois arquivos padrão criados, UTI trainingdata_NI.pat^(^^27^^)^e UTI validationdata_NI.pat,^(^^27^^)^são usados para treinamento e validação da rede, respectivamente. A validação é usada para determinar o desempenho da RNA em padrões que não sejam treinados durante a aprendizagem.^(^^24^^)^

### Simulações de redes neurais

Aqui é usada uma RNA perceptron multicamadas, que é uma rede *feed-forward* sem conexões de atalho. No JavaNNS, em *Tools/Create/Layers* [Ferramentas/Criar/Camadas], a largura é alterada para 12, o número de unidades neuronais de entrada. Novamente, em *Tools/Create/Layers* [Ferramentas/Criar/Camadas], a largura é alterada para 1, o número de unidades de saída. Depois, a rede é conectada em *feed-forward* .^(^^26^^)^A rede resultante é mostrada na figura 1 .

**Figura 1:** A arquitetura da rede neuronal utilizada foi projetada especificamente para este problema no *Java Neural Network Simulator* . O estado da rede aqui mostra uma rede neural artificial treinada com previsão de saída da rede para um dos padrões de validação

A função de inicialização é definida com pesos aleatórios, e o botão *Init* é pressionado, definindo os pesos com valores aleatórios.^(^^26^^)^

### Funções e parâmetros de aprendizagem utilizados

Entre os algoritmos de aprendizagem supervisionada, foram usados os do tipo retropropagação. Há diferentes algoritmos de retropropagação disponíveis no JavaNNS. Os algoritmos retropropagação e retropropagação com *momentum* ^(^^24^^)^são empregados no treinamento dessa rede.

Os valores dos parâmetros usados nas simulações são mostrados na tabela 2 . Esses valores foram obtidos com base nos valores do arquivo xor. README dos exemplos do JavaNNS e nos valores *default* fornecidos no JavaNNS.^(^^24^^,^^26^^)^Os parâmetros do arquivo xor. README são alguns parâmetros que Fischer et al.,^(^^26^^)^usaram para treinar, com êxito, o problema XOR em uma rede neural. A RNA do problema XOR tem algumas semelhanças com a rede construída nessa pesquisa. Fischer et al.,^(^^26^^)^sugeriram o número de ciclos indicado na tabela 2 como necessário para treinar a rede no problema XOR com êxito. Esses parâmetros não foram obtidos com grandes estudos de significância estatística, mas são fornecidos pelo JavaNNS como dicas para que se iniciem as sessões de treinamento.^(^^26^^)^

**Tabela 2:** Valores dos parâmetros usados para todos os modelos de combinações de funções de aprendizagem e conjuntos de parâmetros

Função de aprendizagem:	η	*d*_max_	μ	*c*	Ciclos
Retropropagação com valores do arquivo xor. README	2,0	0,1			2.000
Retropropagação com valores *default*	0,2	0,1			2.000
Retropropagação com *momentum* com valores do arquivo xor. README	3,0	0,1	0,8	0,1	100
Retropropagação com *momentum* com valores *default*	0,2	0,1	0,5	0,1	100

### Simulações

Como existe um componente aleatório no experimento, dez simulações são realizadas para cada um dos algoritmos de aprendizagem e conjuntos de parâmetros, comparando-se os resultados por meio da soma dos erros quadráticos ( *sum of squared errors,* ou SSE) das janelas *Log* e *Error graph* .^(^^26^^)^Os resultados são comparados com parâmetros prévios, bem como entre as diferentes tentativas.

Usando o conjunto de validação, a janela *Error graph* exibe simultaneamente uma linha adicional cor-de-rosa, que corresponde ao erro do conjunto de validação. Utiliza-se a SSE, a soma das diferenças quadráticas entre a saída desejada e a real, para todas as unidades de saída em todos os padrões de treinamento ou validação. A janela de *Log* mostra, durante o treinamento, os valores de SSE dos conjuntos de validação e treinamento.^(^^24^^)^A SSE da janela de *Log* é, então, dividida pelo número de padrões em cada conjunto ( Tabela 3 ).

**Tabela 3:** Erro quadrático médio por padrão para cada uma das funções de aprendizagem e conjuntos de parâmetros, mostrado para cada um dos conjuntos de padrões. Os dados foram obtidos a partir da soma dos erros quadráticos mostrados na janela de *Log* correspondente à última tentativa e dividindo-se pelo número de padrões em cada conjunto

	MSE	
Função de aprendizagem	Conjunto de treinamento	Conjunto de validação
Retropropagação com valores do arquivo xor. README	0,0	0,02760293
Retropropagação com valores *default*	0,0	0,01951062181
Retropropagação com *momentum* com valores do arquivo xor. README	0,0	0,0
Retropropagação com *momentum* com valores *default*	0,0	0,01153508821

MSE: erro médio por padrão.

Após o fim do treinamento, na janela *Updating* , os padrões de treinamento e validação são passados, um a um. Cada padrão é visualizado por cores e numericamente, em todos os neurônios da rede, para observar se a rede teve êxito ou falhou em cada padrão de entrada. A RNA treinada também é avaliada por uma matriz de confusão.

É usada a ordem topológica do modo de atualização.^(^^24^^)^Pressionar *Learn all* na guia de aprendizagem percorre todos os padrões, pelo número de vezes (ciclos) especificado.

Para as dez tentativas, os botões *Init* e *Learn all* são pressionados uma vez para cada tentativa. Os gráficos acumulam-se na janela *Error graph* , em cores diferentes ( Figuras 2 e 3 ). Um fluxograma com um resumo dos métodos é mostrado na figura 4 .

**Figura 2:** Dez simulações realizadas no *Java Neural Network Simulator* com a função de aprendizagem por retropapagação, comparando-se os resultados pela soma dos erros quadráticos na janela *Error graph* . A linha cor-de-rosa corresponde à soma dos erros quadráticos do conjunto de validação, durante a última tentativa. Parte inferior: com os valores de parâmetros do arquivo xor. README. Parte superior: com os valores de parâmetros *default*

**Figura 3:** Dez simulações realizadas no *Java Neural Network Simulator* com a função de aprendizagem por retropropagação com *momentum* , comparando-se os resultados pela soma dos erros quadráticos na janela *Error graph* . A linha cor-de-rosa corresponde à soma dos erros quadráticos do conjunto de validação, durante a última tentativa. À direita: com os valores de parâmetros do arquivo xor. README. À esquerda: com os valores de parâmetros *default*

**Figura 4:** Fluxograma para aprendizagem supervisionada no *Java Neural Network Simulator*

## RESULTADOS

### Função de aprendizagem por retropropagação

O algoritmo de retropropagação teve os seguintes parâmetros: parâmetro de aprendizagem η, indicando a largura do degrau da descida do gradiente, e *dmax* , correspondente à diferença máxima entre uma saída desejada e uma saída real que fosse tolerada.^(^^24^^)^

### Função de aprendizagem por retropropagação com *momentum*

O algoritmo de retropropagação com *momentum* teve os seguintes parâmetros:^(^^24^^)^parâmetro de aprendizagem η, conforme descrito anteriormente; *μ,* termo de *momentum* , indicando a quantidade do ajuste de peso anterior (relativo a 1), adicionado à alteração atual;^(^^24^^)^ *c,* valor de eliminação de *flat spot* , constante adicionada à derivada da função de ativação para que a rede pudesse passar por *flat spots* da superfície do erro^(^^24^^)^e *dmax,* conforme já descrito.

### Tabela de erros e comentários dos resultados

Na figura 2 , parte inferior, a SSE dos padrões de treinamento desapareceu em todas as tentativas antes dos 40 ciclos e muito antes do máximo de 2.000 ciclos. O erro quadrático médio (MSE) do conjunto de validação foi 0,02760293, ou seja, um erro muito pequeno. Em 2.000 ciclos, o valor permaneceu o mesmo, e, nesse MSE, a rede teve seu melhor desempenho, correspondendo ao momento em que o treinamento devia ser interrompido.

Na figura 2 , parte superior, a SSE diminuiu progressivamente, até chegar a 0,0 em todas as tentativas antes dos 450 ciclos. A linha cor-de-rosa atingiu seu MSE mínimo, com erro também muito pequeno. Em comparação à parte inferior da figura 2 , os valores *default* aqui mostraram um desempenho inferior.

Na figura 3 , à direita, a SSE foi 0,0 antes dos dez ciclos, para todas as tentativas. O MSE da linha cor-de-rosa foi 0,0. O algoritmo retropropagação com *momentum* demonstrou um resultado melhor em comparação ao algoritmo retropropagação.

Na figura 3 , à esquerda, a SSE diminuiu progressivamente até chegar a 0,0, em cem ciclos. O MSE da linha cor-de-rosa foi 0,011535088, ou seja, um valor muito pequeno. O desempenho com valores *default* foi inferior ao de valores do arquivo xor. README para esse algoritmo retropropagação com *momentum* . Para os valores de parâmetros *default* , o algoritmo retropropagação com *momentum* mostrou um desempenho superior ao do algoritmo retropropagação.

Na tabela 4 , apresentamos a classificação obtida após treinamento para os 15 padrões de treinamento e os seis de validação, mostrando os êxitos e as falhas. Um padrão foi classificado corretamente como risco de infecções hospitalares presente se a ativação da unidade de saída fosse ≥0,6. Um padrão foi classificado corretamente como risco de infecções hospitalares ausente se a ativação da unidade de saída fosse ≤0,4.

**Tabela 4:** Classificação dos 15 padrões de treinamento e seis de validação, obtidos na aba *Updating* , após treinamento com os padrões de treinamento

Função de aprendizagem	Padrões de treinamento		Padrões de validação	
	Êxitos	Falhas	Êxitos	Falhas
Retropropagação com valores do arquivo xor. README	15	0	6	0
Retropropagação com valores *default*	15	0	6	0
Retropropagação com *momentum* com valores do arquivo xor. README *	15	0	6	0
Retropropagação com *momentum* com valores *default*	15	0	6	0

* Com essa função e esses parâmetros, o erro das unidades foi particularmente pequeno, quase inexistente.

Não houve falhas na categorização de pacientes quanto ao respectivo risco de infecções hospitalares. O algoritmo retropropagação com *momentum* usando valores do arquivo xor. README apresentou erro quase inexistente. A rede teve seu melhor desempenho com o algoritmo de retropropagação com *momentum* , usando-se os valores do arquivo xor. README.

### Avaliação por matriz de confusão

O estado de previsão mais provável para cada padrão foi, então, comparado ao valor real da ativação da unidade de saída para o caso em particular. A matriz de confusão forneceu o número total de casos nas 21 situações. Caso a rede estivesse funcionando bem, então as entradas na diagonal principal seriam grandes, se comparadas às outras fora dessa diagonal. Isso é observado na tabela 5 .

**Tabela 5:** Matriz de confusão de desfechos previstos e efetivamente ocorridos relativos ao risco de infecção hospitalar em 21 casos dos conjuntos de dados de treinamento e validação

Risco de infecções hospitalares		Previsto	
		Risco de infecções hospitalares presente	Risco de infecções hospitalares ausente
**Real**	Risco de infecções hospitalares presente	12	0
	Risco de infecções hospitalares ausente	0	9

## DISCUSSÃO

Os resultados revelaram variações relativas no desempenho das diferentes combinações de algoritmos de aprendizagem e conjuntos de parâmetros. A função de retropropagação com *momentum* demonstrou os melhores resultados. Os resultados foram superiores com os valores de parâmetros do arquivo xor. README. No entanto, como descrito por Fischer et al.,^(^^26^^)^esses parâmetros não devem ser citados como ideais e nem usados em comparações de simuladores de redes.

A habilidade dessa RNA de prever quem tem maior probabilidade de contrair infecções hospitalares é 100% precisa para as características básicas fornecidas. Caso muitos atributos sejam incluídos para caracterização como variáveis de entrada e/ou muitas classes sejam incluídas como variáveis de saída, é necessário incluir uma grande quantidade de padrões. Se, após testar a rede quanto aos êxitos e falhas de cada um dos padrões de treinamento e validação, não for possível alcançar uma alta percentagem de êxitos, talvez seja necessário adicionar uma ou várias camadas de neurônios ocultos. Além disso, outros tipos de funções de aprendizagem por retropropagação podem ser usados no JavaNNS, como os algoritmos de propagação Quickprop ou Resilient.

A distribuição dos fatores de risco para infecções hospitalares difere muito, dependendo do hospital e do tempo. Devido a essas variações, dados de regiões diferentes podem ser de natureza diversa, com diferenças na incidência de infecções hospitalares. Caso não haja um ajuste adequado para a região e o tempo específicos em questão, isso pode levar a falsas conclusões. Recomendam-se usar análises multivariadas retrospectivamente para revelar os fatores de risco associados ao desfecho investigado. Variáveis do tipo *float* também podem ser usadas com o JavaNNS. Na verdade, o uso de dados de entrada contínuos reduz o número de neurônios de entrada e simplifica a arquitetura da rede. Recomenda-se usar o menor número possível de variáveis.^(^^8^^)^

Neste estudo, as infecções hospitalares não foram classificadas por sítio da infecção. Esta pesquisa pode servir de base aos pesquisadores para que eles possam expandir este projeto, e as planilhas podem ser preparadas com classificação por sítio da infecção. Quando o modelo for definido em um contexto de dados reais, ele deve ser avaliado com, pelo menos, um conjunto de validação de padrões de casos reais. Isso precisa ser validado e testado com um número suficiente de padrões de pacientes reais e depende de grandes volumes de dados. Nesse contexto médico, esses conjuntos de dados devem conter centenas ou milhares de amostras de padrões. No caso de um conjunto de dados muito reduzido, um esquema de validação cruzada deve ser usado para validação da rede. Além disso, outros tipos de métodos preditores podem ser testados para comparação dos resultados aos da RNA. O único estudo encontrado na literatura com um contexto clínico semelhante e que contempla algumas dessas limitações é o de Chang et al.^(^^29^^)^

## CONCLUSÃO

Este artigo propõe um método preliminar para prever o risco de infecção hospitalar em unidades de terapia intensiva, adotando-se inteligência artificial e redes neurais artificiais para alcançar melhorias. Esse modelo é usado para demonstrar o desenho e as análises passo a passo, usando-se um conjunto de dados sintéticos derivado do sistema *Japanese Nosocomial Infection Surveillance* . Embora este modelo se baseie em um conjunto de dados sintéticos, o excelente desempenho observado com um pequeno número de padrões sugere que o uso de números maiores de variáveis e camadas de rede, para analisar volumes maiores de dados, pode criar redes neurais artificiais poderosas, possivelmente capazes de prever com precisão o risco de infecção hospitalar em unidades de terapia intensiva.
